# The prevalence of microvascular obstruction in acute myocardial infarction: importance of ST elevation, infarct size, transmurality and infarct age

**DOI:** 10.1186/1532-429X-13-S1-P147

**Published:** 2011-02-02

**Authors:** Lowie MR Van Assche, Sebastiaan CAM Bekkers, Annamalai Senthilkumar, Michele A Parker, Han W Kim, Raymond J Kim

**Affiliations:** 1 Duke University, Durham, NC, USA; 2 Maastricht University, Maastricht, Netherlands

## Objective

To assess the prevalence of microvascular obstruction (MO) by delayed-enhancement cardiac magnetic resonance (DE-CMR) in patients with first acute myocardial infarction (AMI) and describe its relationship with type of infarction, infarct size (IS), transmurality, and infarct age.

## Background

MO has been associated with poor LV remodeling and adverse prognosis. The clinical and CMR characteristics of MO have predominantly been examined in patients with ST-segment elevation MI (STEMI). There are no prior studies that have included both STEMI and non-STEMI patients to allow direct comparison of the prevalence of MO. Additionally, the relationship of MO with IS, transmurality, and infarct age are incompletely understood.

## Methods

We studied 266 consecutive patients from 2 centers (Duke and Maastricht University) with first AMI (elevated biomarkers and angiographically confirmed CAD). Baseline characteristics and the presence of ST-segment elevation (2-contiguous leads ≥0.2 mV in men and ≥0.15 mV in women in leads V2-V3 and/or ≥0.1 mV in other leads) were recorded. Cine and DE-CMR were performed at 4±3 days. IS and transmural extent were measured by planimetry.

## Results

**Figure 1:**
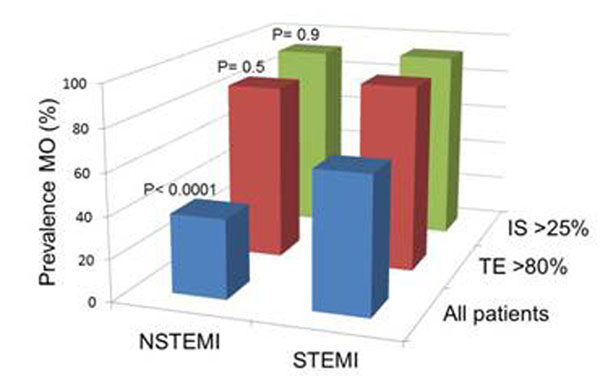
The prevalence of MO for NSTEMI and STEMI in all patients, patients with IS >25% (upper tertile) and patients with transmural extent (TE) >80% (upper tertile).

**Figure 2:**
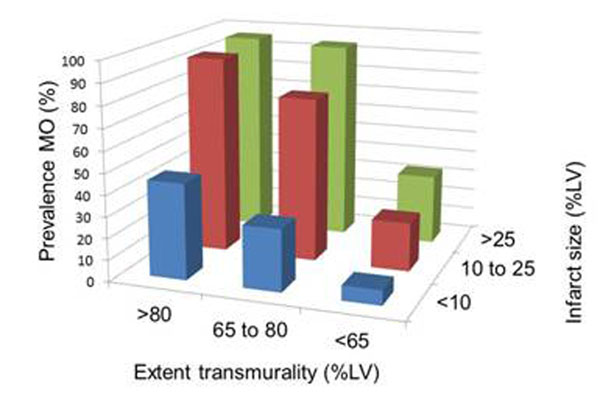
Additive effect of infarct size and transmurality (in tertiles) on the prevalence of MO.

The population consisted of 147 (56%) patients with STEMI, 2 (0.8%) with left bundle branch block, and the remaining 117 (44%) with non-STEMI. The mean age was 59±13 years and 68% were male. The overall observed prevalence of MO was 53%. IS and transmurality were significantly larger in patients with MO than without (24% vs 7%, p<0.0001; and 83% vs 56%, p< 0.0001, respectively). The prevalence of MO was higher in STEMI than non-STEMI (69% vs 31%, p<0.0001, Figure 1), however, when IS was large (upper tertile, >25%) or transmural extent was high (upper tertile, >80%) the prevalence of MO was similar (93% vs 92%, p=0.9; and 90% vs 85%, p=0.5, respectively). The prevalence of MO was dependent on both IS and transmurality (Figure 2). Finally, the prevalence of MO was dependent on infarct age: it was similar over the first 6-days post AMI (days 1-3 =56%, days 4-6 =56%) but decreased over the subsequent week (days 7-9 =42%, days 10-14 =30%). Multivariable analysis demonstrated that only IS, transmurality, and infarct age were independent predictors of MO (p<0.0001, p<0.0001, p=0.05 respectively).

## Conclusions

Microvascular obstruction is more than twice as common in patients with STEMI than non-STEMI, however, the prevalence of MO is similar when accounting for infarct size and transmurality. Only larger infarct size, greater extent of transmurality and earlier infarct age are independent predictors of MO.

